# A Meta-Analysis of *Caspase 9* Polymorphisms in Promoter and Exon Sequence on Cancer Susceptibility

**DOI:** 10.1371/journal.pone.0037443

**Published:** 2012-05-17

**Authors:** Wei Xu, Shengqiang Jiang, Yuanyuan Xu, Bo Chen, Yan Li, Feng Zong, Weihong Zhao, Jianqing Wu

**Affiliations:** 1 Department of Geriatrics, The First Affiliated Hospital of Nanjing Medical University, Nanjing, China; 2 Business School, Hohai University, Nanjing, China; IPO, Inst Port Oncology, Portugal

## Abstract

**Background:**

Caspases are important regulators and executioners in apoptosis pathway and have been defined as either tumor suppressors or oncogenes. Polymorphisms in promoter and exon of *caspase 9* were shown to confer genetic susceptibility to multiple cancers, but the results were inconsistent. To accomplish a more precise estimation of the relationship, a meta-analysis was performed.

**Methodology/Principal Findings:**

We assessed published studies of the association between *caspase 9* polymorphisms and cancer risk from nine studies with 5,528 subjects for rs4645978, six studies with 2,403 subjects for rs105276 and two studies for rs4645981. Overall meta-analysis indicated that no evidence of an association between rs4645978 and cancers was found. Through the stratified analysis, statistically significant reduced cancer risks were observed among Caucasians (AG *vs* AA: OR = 0.81, 95% CI = 0.66–0.99, *P*_heterogeneity_ = 0.150 and the dominant model: OR = 0.86, 95% CI = 0.75–0.99, *P*_heterogeneity_ = 0.290) and prostate cancer. As for rs105276, Ex5+32G>A polymorphism was found with protective effect in overall meta-analysis (AA *vs* GG: OR = 0.75, 95% CI = 0.60–0.92, *P*_heterogeneity_ = 0.887; A *vs* G: OR = 0.85, 95% CI = 0.77–0.95, *P*_heterogeneity_ = 0.739 and the recessive model: OR = 0.68, 95% CI = 0.56–0.82, *P*_heterogeneity_ = 0.309) and Asians group. While for rs4645981, a statistically significant increase in risk of lung cancer was shown in Asians (T *vs* C: OR = 1.23, 95% CI = 1.07–1.42, *P*_heterogeneity_ = 0.399 and the dominant model: OR = 1.22, 95% CI = 1.04–1.43, *P*_heterogeneity_ = 0.660).

**Conclusions/Significance:**

Our meta-analysis suggests that the *caspase 9* rs4645978 most likely contributes to decreased susceptibility to cancer in Caucasians and prostate cancer. The A allele of rs105276 might be a protective factor for cancer, especially for Asians. However, it seems that rs4645981 confers increased susceptibility to lung cancer in Asians.

## Introduction

Evasion of apoptosis is considered to be one of the hallmarks of various human cancers [1]. This cell death modality is executed by caspases (CASPs) and several upstream regulatory factors, which play a crucial role in the development and progression of cancer [2]. CASPs, a family of cysteine-dependent aspartate-specific proteases, can be activated by two distinct but converging pathways: the extrinsic or receptor-mediated pathway and the intrinsic or mitochondrial pathway. Extrinsic (CASP8 and CASP10) and intrinsic (CASP9) initiator CASPs transmit death signals and activate effector CASPs (CASP3, CASP6 and CASP7), which execute a coordinated program of proteolysis, resulting in the destruction of critical cell structure [3].

CASP9, a member of the intrinsic pathway, plays a central role in the mitochondrial apoptotic pathway. The intrinsic or mitochondrial pathway is initiated by the release of cytochrome *c* from mitochondria in response to a variety of cellular stress. Released cytochrome *c* interacts with apoptotic protease activating factor 1 (Apaf-1), procaspase-9 and deoxyadenosine triphosphate (dATP) to form a multiprotein complex called apoptosome. Once bound to the apoptosome, CASP9 is active, which subsequently triggers a cascade of effector CASPs [4]. Some works have shown that CASP 9 is the direct target for regulatory phosphorylation by multiple protein kinases activated in response to extracellular growth/survival factors, osmotic stress or during mitosis [2], which is linked to tumorigenesis and responses to thermotherapy [5].

Recently, several candidate single nucleotide polymorphisms (SNPs) in *CASP9* gene have been reported in public databases (http://www.ncbi.nlm.nih.gov/SNP). It is identified some of these SNPs in the promoter or exon sequence of *CASP9* as potential biomarkers [eg. −1263A>G (rs4645978), −712C>T (rs4645981) and Ex5+32G.>A (rs1052576)], which can modulate susceptibility to cancer, such as those that occur in the lung [6], [7], [8], esophagus [9], stomach [10], [11], colorectal [12], [13], liver [14], pancreas [15], prostate [16], [17], bladder [18], thyroid [19] and hemopoietic system [20], [21]. However, the observed associations of these studies were inconsistent and a single study may be too underpowered to detect the effect of the gene polymorphism on cancer, especially when the sample size is relatively small. Hence, we performed a meta-analysis of all eligible studies to derive more precise estimation of the association of *CASP9* SNPs with cancer risks.

## Materials and Methods

### Publication search

We carried out a search in Medline, Embase, Chinese National Knowledge Infrastructure (CNKI) and Wangfang databases, covering all papers published between 1991 and 2012, with a combination of the following keywords: “caspase 9/caspase-9/CASP9”, “polymorphism/single nucleotide polymorphisms/SNPs/gene susceptibility/genetic variation” and “cancer/tumor/carcinoma/neoplasia/neoplasm” (last search was updated on 10 Mar 2012). We checked potentially relevant publications by examining their titles and abstracts and all studies matching the eligible criteria were retrieved. Besides the database search, the bibliographies of the selected papers and reviews were also examined by hand.

### Criteria for inclusion and exclusion

Studies included in the current meta-analysis had to meet all the following criteria: (a) evaluation of the *CASP9* polymorphisms and cancer risks, (b) use a case-control design, (c) sufficient published data for estimating an odds ratio (OR) with 95% confidence interval (CI). Accordingly, the following exclusion criteria were also used: (a) reviews and repeated literatures, (b) not offering the source of cases and controls and other essential information, (c) not designed as case/control or cohort studies.

### Data extraction

Data were independently abstracted by two investigators (Xu and Li) using a standard protocol and data-collection form according to the criteria listed above. Differences among evaluators were resolved through discussion and rereading with the third investigator (Wu). The following information was extracted from each included study using a standardized data collection protocol (File S1): the surname of first author, publication date, country, ethnicity, cancer, characteristics of control, genotyping methods, total number of cases and controls, and numbers of cases and controls with various *CASP9* genotypes, respectively. Different ethnicity descents were categorised as Caucasian, Asian and Mix (the original studies didn't clarify the race of the subjects or mixed races).

### Statistical methods

OR corresponding to 95% CI was used to assess the strength of association between *CASP9* polymorphism and cancer. The significance of the pooled OR was determined by the *Z*-test, and *P*<0.05 was considered as statistically significant. For *CASP9* rs4645978, the meta-analysis examined the association between G allele and cancer risk compared with that for A allele (G *vs* A); homozygote GG was contrasted with AA and recessive (GG *vs* AA+AG) and dominant (AA+GA *vs* GG) models for allele G were also used, so did rs10525576 and rs4645981. Subgroup analyses were stratified by the study characteristics of racial descent and tumor site, respectively.

Heterogeneity analysis was checked by the chi-square-based *Q*-test [22]. A *P*-value>0.10 for the *Q*-test shows a lack of heterogeneity among the studies, then the pooled OR estimate of each study was calculated by the fixed-effects model (the Mantel-Haenszel method) [23]. Otherwise, the random-effects model (the DerSimonian and Laird method) [24] was used. Hardy-Weinberg equilibrium (HWE) in the control group was estimated by Fisher's exact test and a *P*-value<0.05 was considered significant. Publication bias was assessed by visual inspection of funnel plots in which the standard error of log (OR) of each study was plotted against its log (OR). Funnel plot asymmetry was assessed by the method of Egger's linear regression test. The significance of the intercept was determined by the *t*-test (*P*<0.05 was considered representative of statistically significant publication bias) [25]. Sensitivity analysis was performed by sequential omission of individual studies under various contrasts to reflect the influence of the individual data to the pooled ORs. All of the statistical analyses above were performed with STATA 9.2 (StataCorp, College Station, TX), using two-sided *P*-values.

## Results

### Study characteristics

**Figure 1:**
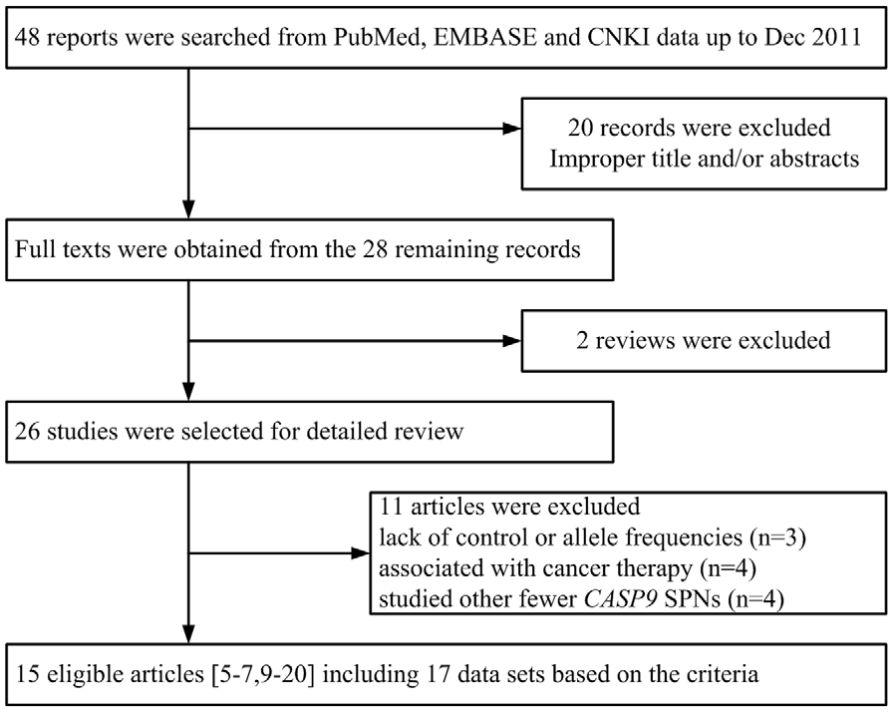
Flow diagram of study identification.

Twenty-eight studies probing the relationship between *CASP9* polymorphisms and cancer susceptibility were identified. During the extraction of data, 13 articles were excluded, because they did not provide allele frequencies needed for OR calculation, the contents were lack of control, their contents mainly associated with cancer therapy, or they studied fewer *CASP9* SPNs (*eg*. rs1052571, 2308941, 4645980), leaving 15 eligible articles [6]–[8], [10]–[21] including 17 data sets based on the search criteria (Fig. 1). Two studies [6], [8] sorted the data about two kinds of *CASP9* polymorphism, that is rs4645978 and rs4645981. Therefore, each group in both studies was considered separately for pooling analysis. The characteristics of selected studies are summarized in Table 1. A total of 9 studies [6], [8], [11], [13], [15]–[19] involving 2,390 cases and 3,138 controls were ultimately analyzed for *CASP9* rs4645978, six studies [7], [10], [12], [14], [20], [21] involving 1,002 cases and 1,401 controls for rs1052576 and two studies [6], [8] for rs4645981. As for *CASP9* rs4645978, there were six subjects of Caucasians [11], [13], [15]–[18] and three subjects of Asians [6], [8], [19]. For rs1052576, there were four groups of Asians [7], [10], [12], [14] and two of Mix [20], [21]. With regard to rs4645981, both studies are about lung cancer in Asians. The controls of all studies mainly came from healthy population and matched for sex and age. All articles used blood samples for genotyping assay. Except for three studies [16]–[18], the distribution of genotypes in the controls was in agreement with HWE.

**Table 1:** Main characteristics of all studies included in the meta-analysis.

Author, year	Country	Ethnicity	Cancer type	Gene type	Genotyping method	No. (cases/controls)		Case(%)			contrlo(%)	
							AA	AG	GG	AA	AG	GG
Park 2006 [5]	Korea	Asian	Lung	rs4645978	PCR-RFLP	432/432	148(34.3)	225(52.0)	59(13.7)	138(32.0)	215(50.0)	79(18.0)
Gangwar 2009 [17]	India	Caucasian	Bladder	rs4645978	PCR-RFLP	212/250	81(38.2)	103(49.0)	28(13.2)	89(36.0)	99(40.0)	62(25.0)
Theodoropoulos 2010 [14]	Greece	Caucasian	Pancreatic	rs4645978	PCR-RFLP	80/160	305(50.2)	3(3.75)	46(57.5)	36(23.0)	91(57.0)	33(21.0)
Kesarwani 2010 [15]	India	Caucasian	Prostate	rs4645978	PCR-RFLP	173/198	82(47.4)	42(24.0)	49(28.3)	70(35.0)	81(41.0)	47(24.0)
Lee 2010 [7]	Korea	Asian	Lung	rs4645978	PCR-RFLP	720/720	260(36.1)	349(49.0)	111(15.4)	269(37.0)	325(45.0)	126(18.0)
Liamarkopoulos2011 [10]	Greece	Caucasian	Gastric	rs4645978	PCR-RFLP	88/480	44(50.0)	34(39.0)	10(11.4)	116(24.0)	239(50.0)	125(26.0)
Theodoropoulos 2011 [12]	Greece	Caucasian	Colorectal	rs4645978	PCR-RFLP	402/480	155(38.6)	181(45.0)	66(16.5)	116(24.0)	239(50.0)	125(26.0)
George 2011 [16]	India	Caucasion	Prostate	rs4645978	PCR-RFLP	165/205	77(46.3)	40(24.0)	48(29.3)	77(46.3)	40(24.0)	48(29.3)
Wang 2011 [18]	China	Asian	Thyroid	rs4645978	PCR-RFLP	118/213	41(34.7)	62(53)	15(12.8)	69(32.4)	93(43.7)	51(23.9)

							GG	GA	AA	GG	GA	AA
Fang 2007 [9]	China	Asian	Gastric	rs1052576	PCR-RFLP	70/100	16(22.9)	39(56.0)	15(21.4)	13(13.0)	42(42.0	45(45.0)
Lan 2007 [19]	USA	Mix	Lymphoma	rs1052576	RT-PCR	455/530	137(30)	223(49.0)	95(21.0)	137(26.0)	260(49.0)	133(25.0)
Lou 2007 [6]	China	Asian	Lung	rs1052576	RT-PCR	81/100	20(24.7)	43(53.0)	18(22.3)	13(13.0)	42(42.0)	45(45.0)
Hosgood 2008 [20]	USA	Mix	Myeloma	rs1052576	RT-PCR	126/511	41(33.0)	65(52.0)	20(67.0)	130(25.0)	252(49.0)	129(25.0)
He 2008 [11]	China	Asian	Colon	rs1052576	RT-PCR	170/100	34(22.0)	95(56.0)	41(24.1)	13(13.0)	42(42.0)	45(45.0)
Wu 2009 [13]	China	Asian	Liver	rs1052576	PCR-RFLP	100/60	43(43.0)	44(44.0)	13(13.0)	25(41.7)	28(46.7)	7(11.7)

							CC	CT	TT	CC	CT	TT
Park 2006 [5]	Korea	Asian	Lung	rs4645981	PCR-RFLP	432/432	216(60.4)	149(35.0)	22(5.1)	298(69.0)	123(29.0)	11(2.6)
Lee 2010 [7]	Korea	Asian	Lung	rs4645981	PCR-RFLP	720/720	438(60.8)	251(35.0)	31(4.3)	482(66.9)	209(29.0)	29(4.0)

### Meta-analysis results

Frequencies of alleles were calculated for controls from the corresponding genotype distribution. The variant alleles had different representations among controls of Asian descent (0.412 for A allele in rs4645978, 0.608 for G allele in rs1052576) and Caucasian descent (0.645 for G allele in rs4645978, 0.498 for A allele in rs1052576).

**Figure 2:**
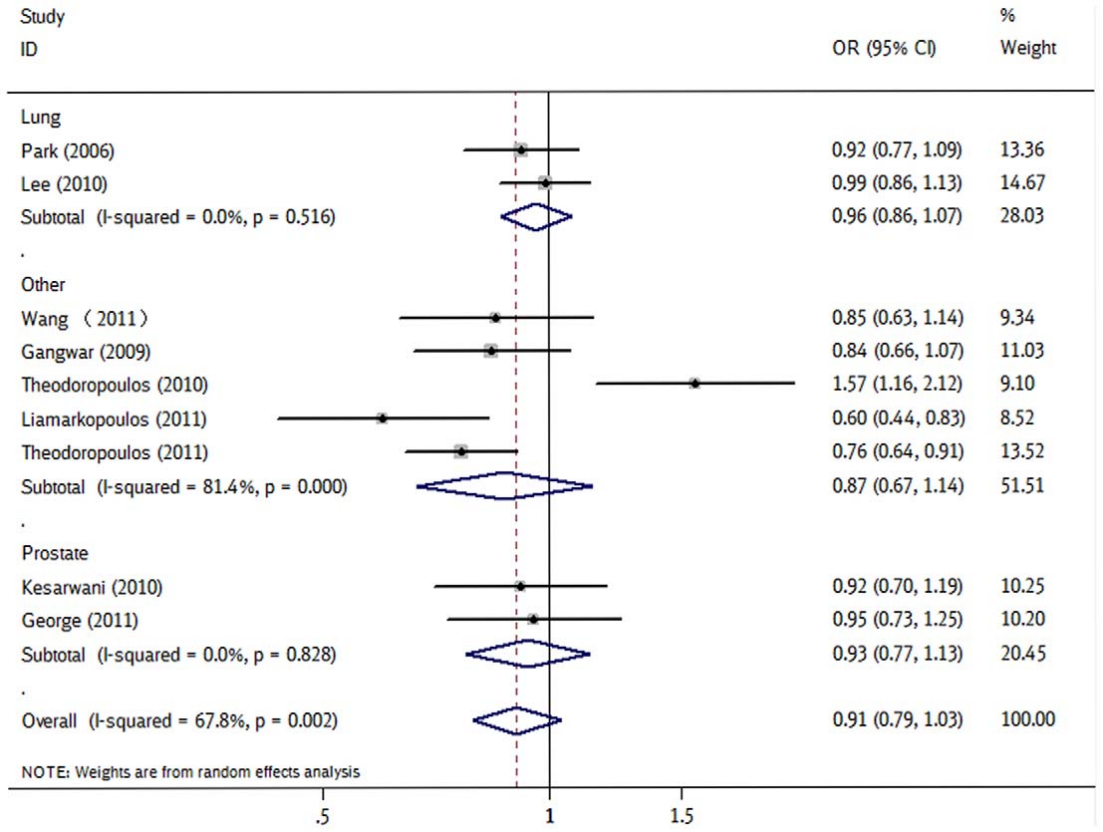
Forest plot of cancer risk associated with rs4645978 under allele contrast in different types of cancers. The squares and horizontal lines correspond to the study-specific OR and 95% CI. The area of the squares reflects the study specific weight (inverse of the variance). The diamond represents the pooled OR and 95% CI.

For *CASP9* rs4645978, five kinds of genetic models did not produce significant association among 9 studies with relatively large heterogeneity (*P*_heterogeneity_ = 0.002–0.096). Through stratified analyses, the heterogeneity of the subgroup significantly reduced. In the stratified analysis by racial descent, statistically significantly decreased cancer risks were found among Caucasian for AG *vs* AA (OR = 0.81, 95% CI = 0.66–0.99, *P*_heterogeneity_ = 0.150, Fig. 2) and the dominant model (OR = 0.86, 95% CI = 0.75–0.99, *P*_heterogeneity_ = 0.290). No significant risks were found among Asians. Carriage of the AG of rs4645978 was found to be associated with protection from prostate cancer (AG *vs* AA: OR = 0.64, 95% CI = 0.47–0.88, *P*_heterogeneity_ = 0.925). No significant associations were observed in lung cancer and other subgroups.

**Figure 3:**
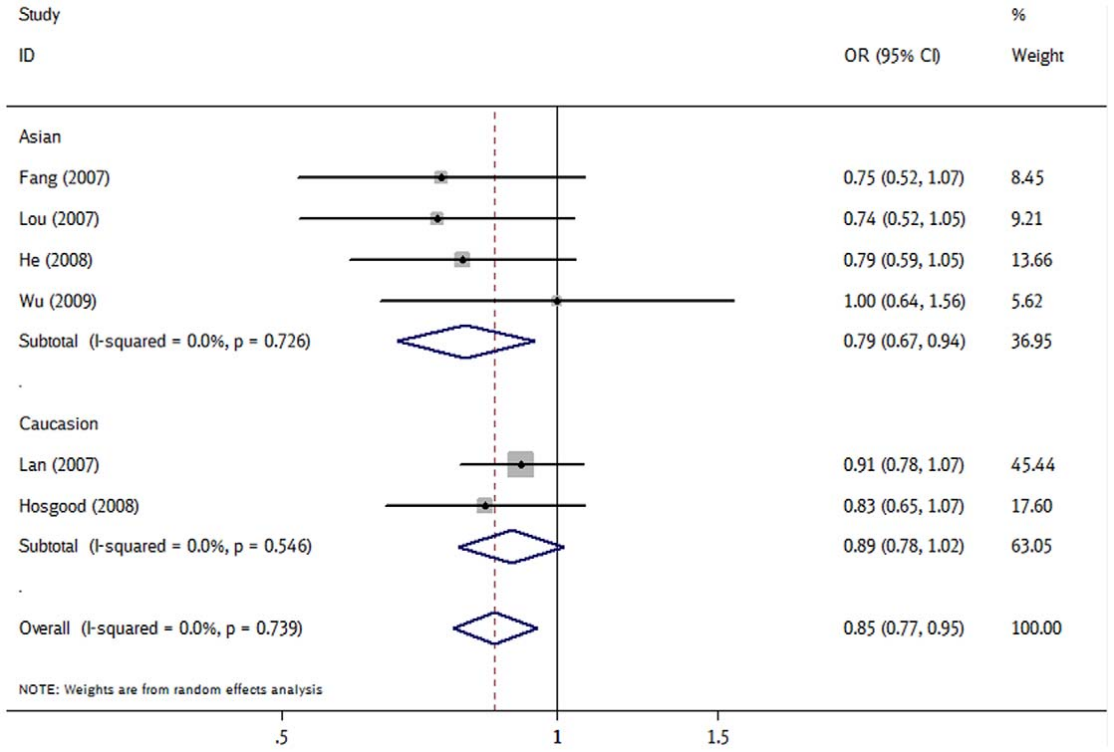
Forest plot of cancer risk associated with rs1052576 under allele contrast (A *vs* G) in different ethnicity. The squares and horizontal lines correspond to the study-specific OR and 95% CI. The area of the squares reflects the study specific weight (inverse of the variance). The diamond represents the pooled OR and 95% CI.

The overall OR with its 95% CI showed statistically association between the *CASP9* rs105276 polymorphism and the reduced risks of cancers (AA *vs* GG: OR = 0.75, 95% CI = 0.60–0.92, *P*_heterogeneity_ = 0.887; A *vs* G: OR = 0.85, 95% CI = 0.77–0.95, *P*_heterogeneity_ = 0.739 and the recessive model: OR = 0.68, 95% CI = 0.56–0.82, *P*_heterogeneity_ = 0.309). In the subgroup analysis by ethnicity, statistically significantly decreased cancer risks were found among Asians for allele contrast (OR = 0.69, 95% CI = 0.49–0.98, *P*_heterogeneity_ = 0.827, Fig. 3), AG *vs* GG (OR = 0.79, 95% CI = 0.67–0.94, *P*_heterogeneity_ = 0.726) and the recessive model (OR = 0.55, 95% CI = 0.40–0.75, *P*_heterogeneity_ = 0.506).

As far as rs4645981, T allele had an effect of increasing the risk of lung cancer in Asians (T *vs* C: OR = 1.23, 95% CI = 1.07–1.42, *P*_heterogeneity_ = 0.399; the dominant model: OR = 1.22, 95% CI = 1.04–1.43, *P*_heterogeneity_ = 0.660). All data are shown in Table 2.

**Table 2:** Main results of pooled ORs and stratification analysis of the rs4645978, rs1052576 and rs4645981 in *caspase 9* on cancer risk.

*Caspase 9*		GG vs AA	AG vs AA	G vs A	Dominant model	Recessive model
rs4645978	N	OR (95% CI) *P*_h_	OR (95% CI) *P*_h_	OR (95% CI) *P*_h_	OR (95% CI) *P*_h_	OR (95% CI) *P*_h_
Total	9	0.80(0.69–1.02)0.006	0.90(0.78–1.04)0.023	0.91(0.79–1.03)0.002	0.92(0.84–1.01)0.096	0.86(0.62–1.17)0.002
Cancer types						
Prostate	2	0.96(0.69–1.35)0.842	0.64(0.47–0.88)0.925	0.94(0.78–1.13)0.828	0.83(0.65–1.05)0.12	1.23(0.89–1.69)0.850
Lung	2	0.88(0.70–1.11)0.465	1.03(0.88–1.19)0.717	0.96(0.86–1.07)0.518	0.99(0.88–1.14)0.987	0.83(0.67–1.03)0.477
Others	5	0.70(0.42–1.15)0.002	0.91(0.74–1.12)0.197	0.87(0.67–1.14)0.000	0.87(0.75–1.01)0.055	0.74(0.40–1.39)0.002
Ethnicity						
Caucasian	5	0.79(0.53–1.19)0.001	0.81(0.66–0.99)0.150	0.96(0.86–1.07)0.002	0.86(0.75–0.99)0.290	0.93(0.64–1.25)0.001
Asian	3	0.85(0.68–1.06)0.465	1.03(0.90–1.18)0.932	0.95(0.86–1.05)0.605	0.99(0.88–1.12)0.331	0.78(0.67–1.03)0.319

*Caspase 9*		AA vs GG	AG vs GG	A vs G	Dominant model	Recessive model
rs1052576	N	OR (95% CI) *P*_h_	OR (95% CI) *P*_h_	OR (95% CI) *P*_h_	OR (95% CI) *P*_h_	OR (95% CI) *P*_h_
Total	6	0.75(0.60–0.92)0.887	0.94(0.80–1.10)1.000	0.85(0.77–0.95)0.739	0.92(0.81–1.05)0.999	0.68(0.56–0.82)0.309
Ethnicity						
Asian	4	0.69(0.49–0.98)0.827	0.94(0.71–1.23)0.997	0.79(0.67–0.94)0.726	0.91(0.73–1.13)0.987	0.55(0.40–0.75)0.506
Mix	2	0.78(0.60–1.03)0.469	0.94(0.78–1.14)0.935	0.89(0.78–1.02)0.546	0.93(0.79–1.09)0.825	0.77(0.60–0.99)0.349

*Caspase 9*		TT vs CC	CT vs TT	T vs C	Dominant model	Recessive model
rs4645981	N	OR (95% CI) *P*_h_	OR (95% CI) *P*_h_	OR (95% CI) *P*_h_	OR (95% CI) *P*_h_	OR (95% CI) *P*_h_
Asian/Lung	2	1.45(0.95–1.21)0.174	1.09(0.71–1.68)0.243	1.23(1.07–1.42)0.399	1.22(1.04–1.43)0.660	1.33(0.87–2.02)0.172

N indicates number of studies involved; Ph: P-value of Q-test for heterogeneity test.

### Publication bias

Begg's funnel plot and Egger's test were performed to assess the publication bias of the literatures. The shapes of the funnel plot for the comparison of the G allele and the A allele of *CASP9* rs4645978 seemed symmetrical in all comparing models. Egger's test was used to provide statistical evidence for funnel plot symmetry. The results still did not suggest any evidence of publication bias (*P* = 0.739 for G over A allele, *P* = 0.776 for GG versus AA, *P* = 0.569 for AG versus AA, *P* = 0.489 for dominant contrast, *P* = 0.752 for reserve contrast, Figure S1). Similarly, no publication bias was detected for association of rs1052576 polymorphisms with cancer (Figure S2).

### Sensitivity analysis

With regard to rs1052576, the results pattern was not impacted by single study in all subgroup studies. As for rs4645978, one study (Theodoropoulos *et al.* [15]) was considered as the main cause of heterogeneity. After exclusion of this study, the heterogeneity no longer existed and the result changed in GG *vs* AA, but others still reached a negative association (Table S1).

## Discussion

Some pioneers of cell death research suggested that pro-apoptotic genes might act as tumor suppressors, whereas oncogenes might fulfill antiapoptotic functions [2], [26]. The efficient of apoptosis in an organism may be the consequence of polymorphisms in any gene regulating or executing apoptosis. Analysis of these polymorphisms may thus help to identify cancer susceptibility, prognosis and tailor treatments accordingly. Growing evidences, including meta-analysis [27] about extrinsic initiator *CASP8*, have suggested that some *CASP* polymorphisms may deregulated the expression and activity of caspase and have attempted to correlate *CASP* polymorphisms with cancer risks.

In current study, we first summarized the data about the association of intrinsic initiator *CASP9* functional polymorphisms and cancer risks. Although many SNPs located in the regions of *CASP9* were identified, most of them were not shown to affect caspase expression and function. However, two SNPs in promoter, *CASP9* −1263A>G (rs4645978) and −712C>T (rs4645981), were observed to alter the transcriptional activity of promoter. An analysis of potential transcription factor-binding sites has shown that the G-C haplotype of −1263A>G and −712C>T polymorphisms have significantly higher transcriptional activity than the A-T haplotype [6], [16], [28]. Therefore, it is possible that “higher production” for *CASP9* in G or C carriers offer protection against the development of some cancers. Our study found a significantly protective effect of *CASP9* G variant in rs4645978 in the subgroups of Caucasians and prostate cancers. Besides, the C allele in rs4645981 also might be a protective factor for lung cancer in Asians.

*CASP9* (Ex5+32G>A, rs1052576) polymorphism encodes for a glutamine to arginine amino acid change at codon 221 of the protein, located at the border of the helix region of *CASP9* [29]. This Q221R variant might lead to conformational changes in the molecule, modify the affinity of this protein to Apaf-1, and eventually influencing carcinogenesis [12], [30]. The current results showed that significant protective association of G/A polymorphism was found in overall meta-analysis and Asian subgroup. It is possible that AA genotype of *CASP9* Ex5+32G>A increases apoptosome activity which provides a protection from various disorders as well as cancer. Due to limited statistical power because of relative small sample size, further evaluation was warranted to confirm these results.

One important property of the gene polymorphisms is that their incidence can vary substantially among different racial populations. In this meta-analysis, significant differences in the prevalence of the *CASP9* rs4645978 A allele and rs1052576 G allele among controls of Asians and Caucasian appeared. In the subgroup analysis by ethnicity, we found the significant association only in one ethnic but not in the other, suggesting genetic diversity between different ethnicities.

In interpreting the results, some cautions should be applied. First, relatively large heterogeneity existed in rs4645978 meta-analysis. Through stratified analyses by tumor site and racial descent respectively, heterogeneity reduced significantly. Therefore, we presumed that the relatively large heterogeneity mainly results from differences of ethnicity and tumor types. Simultaneously, the heterogeneity might also be caused by the differences in the selection of controls, age distribution and lifestyle factors. Moreover, lack of the original data of the reviewed studies limited our further evaluation of potential interactions because the interactions between gene-to-gene, gene-to-environment, and even different polymorphic loci of the same gene may modulate cancer risk. Finally, a relatively limited number of studies and samples was analyzed for our assessment. This was in part because the selected original studies were performed with rather limited sample sets. Also, because the reports included in our meta-analysis were limited to those published in either English or Chinese, it is possible that some relevant published and unpublished studies, likely to have null results, were not included, possibly biasing the result, although the result for publication bias was not statistically significant.

In summary, this meta-analysis supports that the *caspase 9* rs4645978 most likely contributes to decreased susceptibility to cancer in Caucasians and prostate cancer. For rs105276, the A allele might be a protective factor for cancer, especially for Asians. However, it seems that rs4645981 confers increased susceptibility to lung cancer in Asians.

## Supporting Information

Figure S1 Funnel plot of publication bias in rs4645978 studies. Log OR is plotted versus standard error for each of studies in this meta-analysis. Each point represents a separate study for the indicated association in all comparing models.
(DOC)

Figure S2 Funnel plot of publication bias in rs105276 studies. Each point represents a separate study for the indicated association.
(DOC)

Table S1 ORs (95% CI) of sensitivity analysis for rs4645978 and rs105276.
(DOC)

File S1 MOOSE Checklist and Flowchart for the meta-analysis.
(DOC)

## References

[1] Hanahan D, Weinberg RA (2011). Hallmarks of cancer: the next generation.. Cell.

[2] Olsson M, Zhivotovsky B (2011). Caspases and cancer.. Cell Death Differ.

[3] Degterev A, Boyce M, Yuan J (2003). A decade of caspases.. Oncogene.

[4] Adrain C, Martin SJ (2001). The mitochondrial apoptosome: a killer unleashed by the cytochrome seas.. Trends Biochem Sci.

[5] Allan LA, Clarke PR (2009). Apoptosis and autophagy: Regulation of caspase-9 by phosphorylation.. FEBS J.

[6] Park JY, Park JM, Jang JS, Choi JE, Kim KM (2006). Caspase 9 promoter polymorphisms and risk of primary lung cancer.. Hum Mol Genet.

[7] Lou Y, Fang CQ, Li JH (2007). A study on the expression of CASP9 gene and its polymorphism distribution in non-small cell lung cancer.. Zhonghua Yi Xue Yi Chuan Xue Za Zhi.

[8] Lee SY, Choi YY, Choi JE, Kim MJ, Kim JS (2010). Polymorphisms in the caspase genes and the risk of lung cancer.. J Thorac Oncol.

[9] Liu CY, Wu MC, Chen F, Ter-Minassian M, Asomaning K (2010). A Large-scale genetic association study of esophageal adenocarcinoma risk.. Carcinogenesis.

[10] Fang CQ, Liu SL, Lou Y, Li JH (2007). Expression of the caspase 9 gene and its polymorphism distribution in gastric cancer.. Shijie Huaren Xiaohua Zazhi.

[11] Liamarkopoulos E, Gazouli M, Aravantinos G, Tzanakis N, Theodoropoulos G (2011). Caspase 8 and caspase 9 gene polymorphisms and susceptibility to gastric cancer.. Gastric Cancer.

[12] He XM, Wang LL, Fang CQ, Liu SL, Lou Y (2008). Expression of CASP9 gene and its polymorphism distribution in colon cancer.. Shijie Huaren Xiaohua Zazhi.

[13] Theodoropoulos GE, Gazouli M, Vaiopoulou A, Leandrou M, Nikouli S (2011). Polymorphisms of Caspase 8 and Caspase 9 gene and colorectal cancer susceptibility and prognosis.. Int J Colorectal Dis.

[14] Wu H (2009). Correlation between Caspase 9 single nucleotide polymorphism and susceptibility to hepatocellular carcinoma in Fusui county of Guangxi..

[15] Theodoropoulos GE, Michalopoulos NV, Panoussopoulos SG, Taka S, Gazouli M (2010). Effects of caspase-9 and survivin gene polymorphisms in pancreatic cancer risk and tumor characteristics.. Pancreas.

[16] Kesarwani P, Mandal RK, Maheshwari R, Mittal RD (2011). Influence of caspases 8 and 9 gene promoter polymorphism on prostate cancer susceptibility and early development of hormone refractory prostate cancer.. BJU Int.

[17] George GP, Mandal RK, Kesarwani P, Sankhwar SN, Mandhani A (2011). Polymorphisms and haplotypes in caspases 8 and 9 genes and risk for prostate cancer: A case-control study in cohort of North India.. Urol Oncol.

[18] Gangwar R, Mandhani A, Mittal RD (2009). Caspase 9 and caspase 8 gene polymorphisms and susceptibility to bladder cancer in north Indian population.. Ann Surg Oncol.

[19] Wang YX, Zhao L, Wang XY, Liu CM, Yu SG (2011). Role of Caspase 8, Caspase 9 and Bcl-2 polymorphisms in papillary thyroid carcinoma risk in Han Chinese population.. Med Oncol.

[20] Lan Q, Zheng T, Chanock S, Zhang Y, Shen M (2007). Genetic variants in caspase genes and susceptibility to non-Hodgkin lymphoma.. Carcinogenesis.

[21] Hosgood HD, Baris D, Zhang Y, Zhu Y, Zheng T (2008). Caspase polymorphisms and genetic susceptibility to multiple myeloma.. Hematol Oncol.

[22] Cochran WG (1954). The combination of estimates from different experiments.. Biometrics.

[23] Mantel N, Haenszel W (1959). Statistical aspects of the analysis of data from retrospective studies of disease.. J Natl Cancer Inst.

[24] DerSimonian R, Laird N (1986). Meta-analysis in clinical trials.. Control Clin Trials.

[25] Egger M, Davey Smith G, Schneider M, Minder C (1997). Bias in meta-analysis detected by a simple, graphical test.. BMJ.

[26] Ghavami S, Hashemi M, Ande SR, Yeganeh B, Xiao W (2009). Apoptosis and cancer: mutations within caspase genes.. J Med Genet.

[27] Yin M, Yan J, Wei S, Wei Q (2010). CASP8 polymorphisms contribute to cancer susceptibility: evidence from a meta-analysis of 23 publications with 55 individual studies.. Carcinogenesis.

[28] Grabe N (2002). AliBaba2: context specific identification of transcription factor binding sites.. In Silico Biol.

[29] Hirano A, Nagai H, Harada H, Haga S, Kajiwara T (2001). Two novel single-nucleotide polymorphisms of the Caspase-9 (CASP9) gene in the Japanese population.. Genes Immun.

[30] Andreoli V, Trecroci F, La Russa A, Valentino P, Condino F (2009). CASP-9: A susceptibility locus for multiple sclerosis in Italy.. J Neuroimmunol.

