# Supplementary material for: A Meta-Analysis of Caspase 9 Polymorphisms in Promoter and Exon Sequence on Cancer Susceptibility
Source: PLoS One. 2012 May 17;7(5):e37443. doi: 10.1371/journal.pone.0037443 (PMC3355128; doi:10.1371/journal.pone.0037443)
Supplement: Figure S1 — Funnel plot of publication bias in rs4645978 studies. Log OR is plotted versus standard error for each of studies in this meta-analysis. Each point represents a separate study for the indicated association in all comparing models. (DOC) [file pone.0037443.s001.doc]

Funnel plot of publication bias in rs4645978 studies. Log OR is plotted versus standard error for each of studies in this meta-analysis. Each point represents a separate study for the indicated association by G over A allele (*t* = 0.35, df = 8, *P* = 0.739).

Funnel plot of publication bias in rs4645978 studies. Each point represents a separate study for the indicated association by GG versus AA (*t* = 0.30, df = 8, *P* = 0.776).

Funnel plot of publication bias in rs4645978 studies. Each point represents a separate study for the indicated association by AG versus AA (*t* = 0.60, df = 8, *P* = 0.569).

Funnel plot of publication bias in rs4645978 studies. Each point represents a separate study for the indicated association by dominant contrast (*t* = 0.22, df = 8, *P* = 0.489).

Funnel plot of publication bias in rs4645978 studies. Each point represents a separate study for the indicated association by reserve contrast (*t* = 0.33, df = 8, *P* = 0.752).
