# Supplementary material for: A Meta-Analysis of Caspase 9 Polymorphisms in Promoter and Exon Sequence on Cancer Susceptibility
Source: PLoS One. 2012 May 17;7(5):e37443. doi: 10.1371/journal.pone.0037443 (PMC3355128; doi:10.1371/journal.pone.0037443)
Supplement: Figure S2 — Funnel plot of publication bias in rs105276 studies. Each point represents a separate study for the indicated association. (DOC) [file pone.0037443.s002.doc]

Funnel plot of publication bias in rs1052576 studies. Log OR is plotted versus standard error for each of studies in this meta-analysis. Each point represents a separate study for the indicated association by A over G allele (*t* = 0.31, df = 5, *P* = 0.769).

Funnel plot of publication bias in rs1052576 studies. Each point represents a separate study for the indicated association by AA versus GG (*t* = 0.75, df = 5, *P* = 0.497).

Funnel plot of publication bias in rs1052576 studies. Each point represents a separate study for the indicated association by AG versus GG (*t* = 0.56, df = 5, *P* = 0.603).

Funnel plot of publication bias in rs1052576 studies. Each point represents a separate study for the indicated association by dominant contrast (*t* = 1.01, df = 5, *P* = 0.371).

Funnel plot of publication bias in rs1052576 studies. Each point represents a separate study for the indicated association by reserve contrast (*t* = 0.95, df = 5, *P* = 0.394).
