# Supplementary material for: A Meta-Analysis of Caspase 9 Polymorphisms in Promoter and Exon Sequence on Cancer Susceptibility
Source: PLoS One. 2012 May 17;7(5):e37443. doi: 10.1371/journal.pone.0037443 (PMC3355128; doi:10.1371/journal.pone.0037443)
Supplement: Table S1 — ORs (95% CI) of sensitivity analysis for rs4645978 and rs105276. (DOC) [file pone.0037443.s003.doc]

| **Table S1**: ORs (95% CI) of sensitivity analysis for rs4645978 and rs1052576. | | | | | |
| --- | --- | --- | --- | --- | --- |
| Excluding literature | GG *vs* AA | AG *vs* AA | G *vs* A | Dominant model | Recessive model |
| one by one | OR (95% CI) P*h* | OR (95% CI) P*h* | OR (95% CI) P*h* | OR (95% CI) P*h* | OR (95% CI) P*h* |
| All for 4645978 | 0.80(0.69-1.02)0.006 | 0.90(0.78-1.04)0.023 | 0.91(0.79-1.03)0.002 | 0.92(0.83-1.01)0.180 | 0.86(0.62-1.17)0.002 |
| Park 2006 | 0.80(0.60-1.07)0.003 | 0.88(0.74-1.04)0.072 | 0.90(0.77-1.06)0.001 | 0.91(0.81-1.02)0.132 | 0.87(0.60-1.25)0.001 |
| Gangwar 2009 | 0.81(0.70-1.05)0.005 | 0.88(0.75-1.03)0.080 | 0.88(0.75-1.03)0.080 | 0.91(0.82-1.02)0.125 | 0.91(0.65-1.27)0.001 |
| Theodoropoulos 2010 | 0.74(0.60-0.91)0.112 | 0.88(0.76-1.02)0.097 | 0.88(0.76-1.02)0.097 | 0.92(0.83-1.01)0.180 | 0.76(0.60-1.03)0.017 |
| Kesarwani 2010 | 0.78(0.59-1.03)0.004 | 0.93(0.81-1.07)0.164 | 0.93(0.81-1.07)0.164 | 0.93(0.83-1.03)0.154 | 0.82(0.58-1.16)0.001 |
| Lee 2010 | 0.77(0.58-1.04)0.006 | 0.87(0.74-1.02)0.141 | 0.87(0.74-1.02)0.141 | 0.89(0.80-1.00)0.241 | 0.85(0.58-1.25)0.002 |
| Liamarkopoulos 2011 | 0.85(0.67-1.07)0.024 | 0.93(0.81-1.07)0.150 | 0.93(0.81-1.07)0.150 | 0.94(0.86-1.02)0.367 | 0.91(0.66-1.26)0.001 |
| Theodoropoulos 2011 | 0.84(0.64-1.10)0.016 | 0.92(0.78-1.08)0.105 | 0.95(0.85-1.06)0.105 | 0.94(0.85-1.04)0.251 | 0.89(0.63-1.27)0.001 |
| George 2011 | 0.77(0.59-1.02)0.004 | 0.92(0.82-1.06)0.140 | 0.92(0.80-1.06)0.140 | 0.92(0.83-1.03)0.139 | 0.81(0.58-1.14)0.001 |
| Wang 2011 | 0.81(0.62-1.07)0.004 | 0.91(0.82-1.01)0.072 | 0.88(0.76-1.03)0.072 | 0.91(0.82-1.02)0.125 | 0.90(0.64-1.25)0.001 |
| Excluding literature | AA *vs* GG | GA *vs* GG | A *vs* G | Dominant model | Recessive model |
| one by one | OR (95% CI) P*h* | OR (95% CI) P*h* | OR (95% CI) P*h* | OR (95% CI) P*h* | OR (95% CI) P*h* |
| All for rs1052576 | 0.75(0.60-0.92)0.887 | 0.94(0.80-1.10)1.000 | 0.85(0.77-0.95)0.739 | 0.92(0.81-1.05)0.999 | 0.68(0.56-0.82)0.309 |
| Fang 2007 | 0.76(0.61-0.95)0.834 | 0.94(0.80-1.11)1.000 | 0.76(0.61-0.95)0.834 | 0.93(0.81-1.06)0.996 | 0.70(0.57-0.86)0.314 |
| Lan 2007 | 0.94(0.80-1.00)0.999 | 0.93(0.75-1.16)1.000 | 0.81(0.70-0.93)0.842 | 0.91(0.76-1.08)0.998 | 0.57(0.44-0.75)0.641 |
| Lou 2007 | 0.73(0.59-0.91)0.872 | 0.94(0.80-1.11)1.000 | 0.87(0.78-0.97)0.735 | 0.93(0.81-1.07)0.998 | 0.70(0.57-0.86)0.303 |
| Hosgood 2008 | 0.68(0.51-0.91)0.992 | 0.94(0.79-1.12)1.000 | 0.85(0.77-0.95)0.739 | 0.93(0.80-1.07)0.995 | 0.69(0.55-0.85)0.209 |
| He 2008 | 0.76(0.61-0.96)0.832 | 0.94(0.79-1.11)1.000 | 0.86(0.77-0.97)0.663 | 0.92(0.80-1.06)0.994 | 0.71(0.57-0.87)0.297 |
| Wu 2009 | 0.78(0.69-0.97)0.842 | 0.94(0.80-1.10)1.000 | 0.85(0.76-0.94)0.692 | 0.92(0.80-1.05)0.997 | 0.66(0.54-0.81)0.294 |
| P*h*: P-value of Q-test for heterogeneity test. | | |  |  |  |
