# Supplementary material for: A Meta-Analysis of Caspase 9 Polymorphisms in Promoter and Exon Sequence on Cancer Susceptibility
Source: PLoS One. 2012 May 17;7(5):e37443. doi: 10.1371/journal.pone.0037443 (PMC3355128; doi:10.1371/journal.pone.0037443)
Supplement: File S1 — MOOSE Checklist and Flowchart for the meta-analysis. (DOC) [file pone.0037443.s004.doc]

**MOOSE Checklist of the Current Meta-analysis**

**Reporting of background should include**

Problem definition: Effects of *caspase 9* polymorphisms in promoter and exon sequence on cancer susceptibility

Hypothesis statement: *caspase 9* rs4645978, rs105276 and rs4645981 polymorphisms contribute to cancer risk

Description of study outcome: The *caspase 9* rs4645978 most likely contributes to decreased susceptibility to cancer in Caucasians and prostate cancer. The A allele of rs105276 might be a protective factor for cancer, especially for Asians. However, it seems that rs4645981 confers increased susceptibility to lung cancer in Asians.

Type of exposure or intervention used: *caspase 9* rs4645978, rs105276 and rs4645981 polymorphisms

Type of study designs used: Meta-analysis

Study population: Cancer patients and healthy controls

**Reporting of search strategy should include**

Qualifications of searchers: Dr. Xu and Chen

Search strategy, including time period include in the synthesis and keywords: P3 publication search

Effort to include all available studies, including contact with authors: Yes

Databases and registries searched: P3 publication search

Search software used, name and version, including special features used: PubMed, EMBASE and CNKI

Use of hand searching: Yes

List of citations located and those excluded, including justification: Figure 1 and Table 1

Method of addressing articles published in languages other than English: Translation software

Method of handing abstracts and unpublished studies: Searched, but not found

Description of any contact with authors: None

**Reporting of methods should include**

Description of relevance or appropriateness of studies assembled for assessing the hypothesis to be tested: Table 1

Rationale for the selection and coding of data: (a) evaluation of the *CASP9* polymorphisms and cancer risks, (b) use a case-control design, (c) sufficient published data for estimating an odds ratio (OR) with 95% confidence interval (CI).

Documentation of how data were classified and coded: Two investigators (Xu and Chen) used a standard protocol and data-collection form. They discussed with Pro. Wu, then decide the data.

Assessment of confounding: Ditto

Assessment of study quality, including binding of quality assessors; stratification or regression on possible predictors of study results: Table 1, 2

Assessment of heterogeneity: The chi-square-based *Q*-test

Description of statistical methods in sufficient detail to be replicated: A *P*_heterogeneity_ > 0.10 for the *Q*-test indicates a lack of heterogeneity among the studies, then the pooled OR estimate of each study was calculated by the fixed-effects model (the Mantel-Haenszel method). Otherwise, the random-effects model (the DerSimonian and Laird method)

Provision of appropriate tables and graphics: Table 2 and Figure 2, 3

**Reporting of results should include**

Graphic summarizing individual study estimates and overall estimate: Table 2

Table giving descriptive information for each study included: Table 2

Results of sensitivity testing: P7

Indication of statistical uncertainty of findings: P7

**Reporting of discussion should include**

Quantitative assessment of bias: P7

Justification for exclusion: P8, P9

Assessment of quality of included studies: P8, P9

**Reporting of conclusions should include**

Consideration of alternative explanations for observed results: P9

Generalization of the conclusions: P10

Guidelines for future research: P10

Disclosure of funding source: This work was supported by a grant from the National Natural Science Foundation of China (No. 30971320).
